# Central venous pressure estimation with force-coupled ultrasound of the internal jugular vein

**DOI:** 10.1038/s41598-022-22867-w

**Published:** 2023-01-27

**Authors:** Alex Jaffe, Ivan Goryachev, Charles Sodini, Brian W. Anthony

**Affiliations:** Massachusetts Institute of Technology, Cambridge, USA

**Keywords:** Cardiology, Ultrasonography

## Abstract

We estimate central venous pressure (CVP) with force-coupled ultrasound imaging of the internal jugular vein (IJV). We acquire ultrasound images while measuring force applied over the IJV by the ultrasound probe imaging surface. We record collapse force, the force required to completely occlude the vein, in 27 healthy subjects. We find supine collapse force and jugular venous pulsation height (JVP), the clinical noninvasive standard, have a linear correlation coefficient of r^2^ = 0.89 and an average absolute difference of 0.23 mmHg when estimating CVP. We perturb our estimate negatively by tilting 16 degrees above supine and observe decreases in collapse force for every subject which are predictable from our CVP estimates. We perturb venous pressure positively to values experienced in decompensated heart failure by having subjects perform the Valsalva maneuver while the IJV is being collapsed and observe an increase in collapse force for every subject. Finally, we derive a CVP waveform with an inverse three-dimensional finite element optimization that uses supine collapse force and segmented force-coupled ultrasound data at approximately constant force.

Congestive Heart Failure (CHF) is a clinical syndrome in which the heart’s pumping ability decreases, leading to fluid buildup in the circulatory system^1^. It currently affects about 6.2 million people and is recorded on about 380,000 death certificates annually in the United States^2^. In heart failure progression, this fluid buildup, which increases venous pressure, begins as the circulatory system’s way of compensating for a decreased pumping ability in order to maintain adequate cardiac output to perfuse the organs with oxygen^1^. Decompensated heart failure occurs when the increase in fluid volume is no longer able to maintain cardiac output, initiating a vicious cycle of continually increasing fluid volume despite eventually detrimental effects to cardiac output^1,3^. At this point, venous pressure is high enough to release fluid from the circulatory system, causing peripheral and pulmonary edema, which can lead to death^1,4,5^.

Treatment for elevated venous pressure in decompensated heart failure involves administration of diuretics informed by central venous pressure (CVP) measurement^6^. The gold standard for CVP assessment is direct measurement of pressure in the superior vena cava or right atrium with catheterization^7,8^. However, noninvasive assessment is often chosen unless the patient is already catheterized^6,8^. The standard noninvasive venous pressure measurements are jugular venous pulsation height (JVP) measurement and inferior vena cava diameter measurement^9,10^. Although rooted in physiological principles, these methods fall short of achieving a reliable noninvasive standard to guide treatment decisions compared to their arterial pressure counterpart, the brachial blood pressure cuff, which has a methodology not replicable in veins^10–13^. There have been several attempts to improve venous pressure assessment in recent years, especially in the field of medical ultrasound^14–18^.

Force-coupled ultrasound is a technique which combines ultrasound imaging with simultaneous measurement of the force applied by the imaging surface of the ultrasound probe to the skin over the image plane^3,19,20^. This technique has had moderate success in the accurate estimation of blood pressure in the carotid artery in which the carotid artery is never close to being fully collapsed^21–23^. Additionally, a force-coupled single-element ultrasound study shows that changes in mean jugular venous pressure can be sensed by measuring the force necessary to completely occlude the internal jugular vein (IJV)^24^. A Korotkoff blood pressure cuff (sphygmomanometer) estimates systolic and diastolic brachial blood pressure by recording the maximum (systolic) and minimum (diastolic) cuff pressures at which the sound of artery wall collapse action occurs^25^. While acknowledging that symmetric application of external pressure to the IJV is not feasible, we believe asymmetric compression of the IJV with force-coupled ultrasound imaging should be studied as a venous analog to the Korotkoff cuff^1^. The IJV is of lower pressure and stiffness than any of its surrounding anatomical landmarks, which should allow for its compression at a lower force than what would compress other surrounding tissue. Additionally, since there is no valve in between the IJV at the base of the neck and the right atrium, the pressure waveform of the right atrium should be reflected in that of the IJV^1^.

For this preliminary validation study of our force-coupled ultrasound technique to measure the collapse force of the IJV, we do not have a gold standard comparison. Therefore, we compare force-coupled ultrasound compression of the IJV to a quantitative version of the noninvasive standard JVP measurement in the same subjects in the supine position and when elevated to a small angle above supine. We have healthy subjects perform the Valsalva maneuver to artificially increase venous pressure to the range measured during decompensated heart failure^1,26^. Finally, we use the correlation of supine collapse forces to JVP measurements and force-coupled ultrasound IJV area measurements to derive a jugular venous pressure waveform in an effort to identify components of the right atrial pressure waveform. Our central aim is to demonstrate our technique has similar accuracy to JVP in the small range of venous pressures we can compare in healthy individuals. We also hope to display our method’s potential to provide accurate and information-rich measurements for the full range of conceivable venous pressures which could be observed, where the noninvasive standard JVP falters.

## Results

We utilize a force-coupled ultrasound probe to simultaneously obtain ultrasound images of the IJV while recording the force applied by the imaging surface of the ultrasound probe. Supplementary Fig. 1 and Supplementary Table 1 describe the force-coupling in detail. Supplementary Fig. 2 shows details of the ultrasound and LabVIEW interfaces regarding acquiring high quality short-axis cross-section images of the IJV under compression such that post-processing can proceed in an automated fashion.

**Figure 1:**
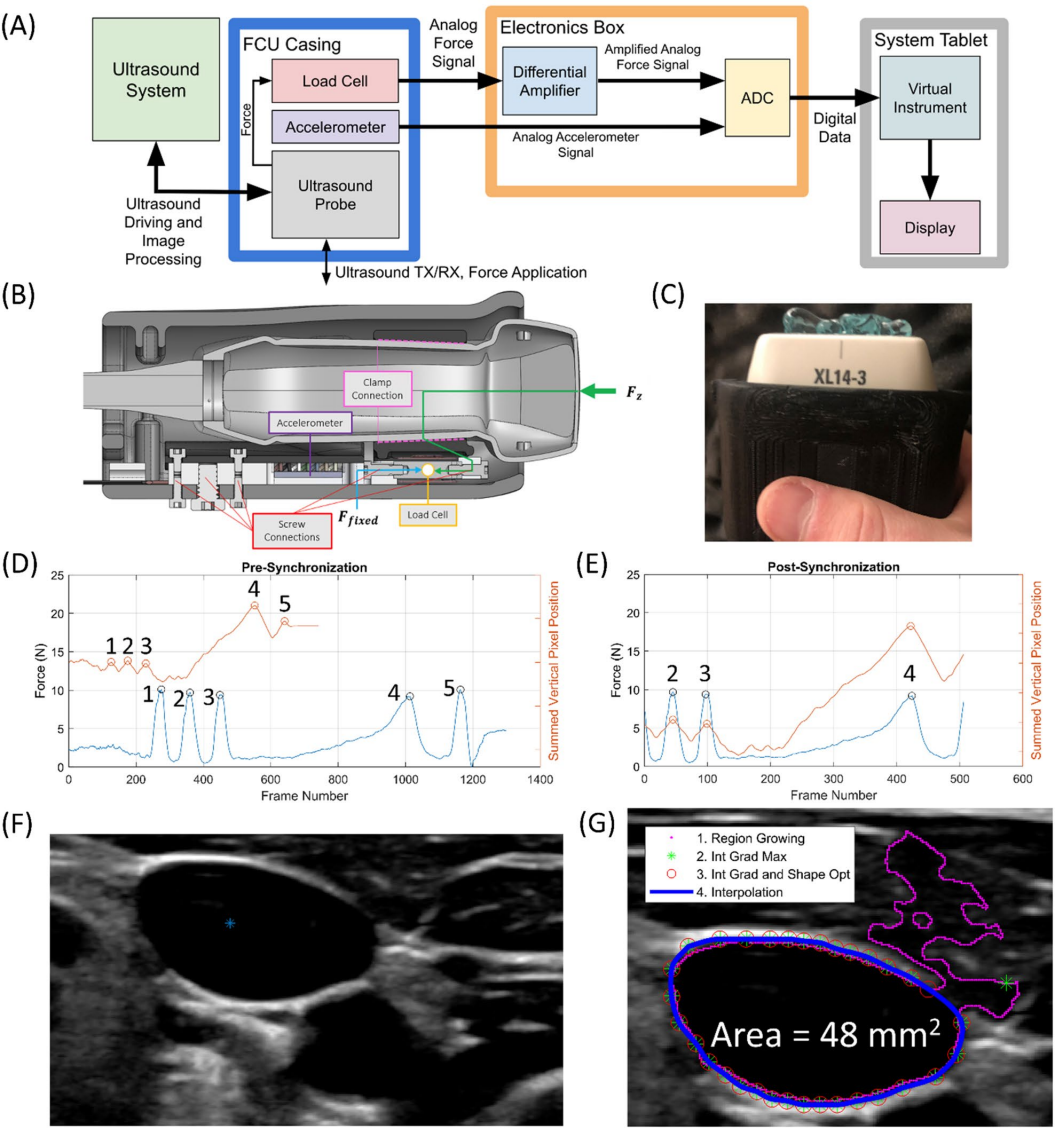
(**A**) Force-coupled ultrasound data collection block diagram. (**B**) CAD diagram of force-coupled ultrasound probe longitudinal cross-section. The force transmission is shown in green from the imaging surface of the ultrasound probe to the clamp connection to the load cell. (**C**) Picture of handheld probe with ultrasound gel on it. (**D**) Force and optical flow overlay pre-synchronization. Force is in blue while optical flow is in orange. Synchronization-relevant peaks are marked. (**E**) Force and optical flow overlay post-synchronization. Force is in blue while optical flow is in orange. Synchronization-relevant peaks are marked. (**F**) IJV detection with seed point indicated by blue asterisk in subject 2. (**G**) Segmentation of an open IJV with all intermediate steps included in subject 2. The pink indicates the first stage region growing output. The green asterisks represent the second stage radial line intensity gradient maximization output. The red circles represent the third stage intensity gradient and shape optimization. The blue tracing represents the final 2000-point interpolation stage.

A data acquisition system block diagram, a probe force transmission diagram, and a picture of the probe with ultrasound gel are depicted in Fig. 1A–C. For each sequence of images of the short axis cross-section of the left IJV acquired with a force-coupled ultrasound probe, we must (1) synchronize to assign a force to each ultrasound image, (2) detect the IJV in one image we seek to analyze, and (3) segment the IJV in each image we seek to analyze. The fully automated post-processing is further described in Fig. 1D–G and mirrors what was done in our previous carotid study^23,27^. Supplementary Table 2 and Supplementary Fig. 3 describe the IJV detection portion of the post-processing in more detail.

Our study involves obtaining force-coupled ultrasound data on the left internal jugular veins of 27 healthy volunteers. The MIT Institutional Review Board approved this study under protocol number 2007000193. Informed consent was obtained for all subjects of the study. All data collection is noninvasive and is carried out in accordance with subject anonymity and minimal risk practices.

**Figure 2:**
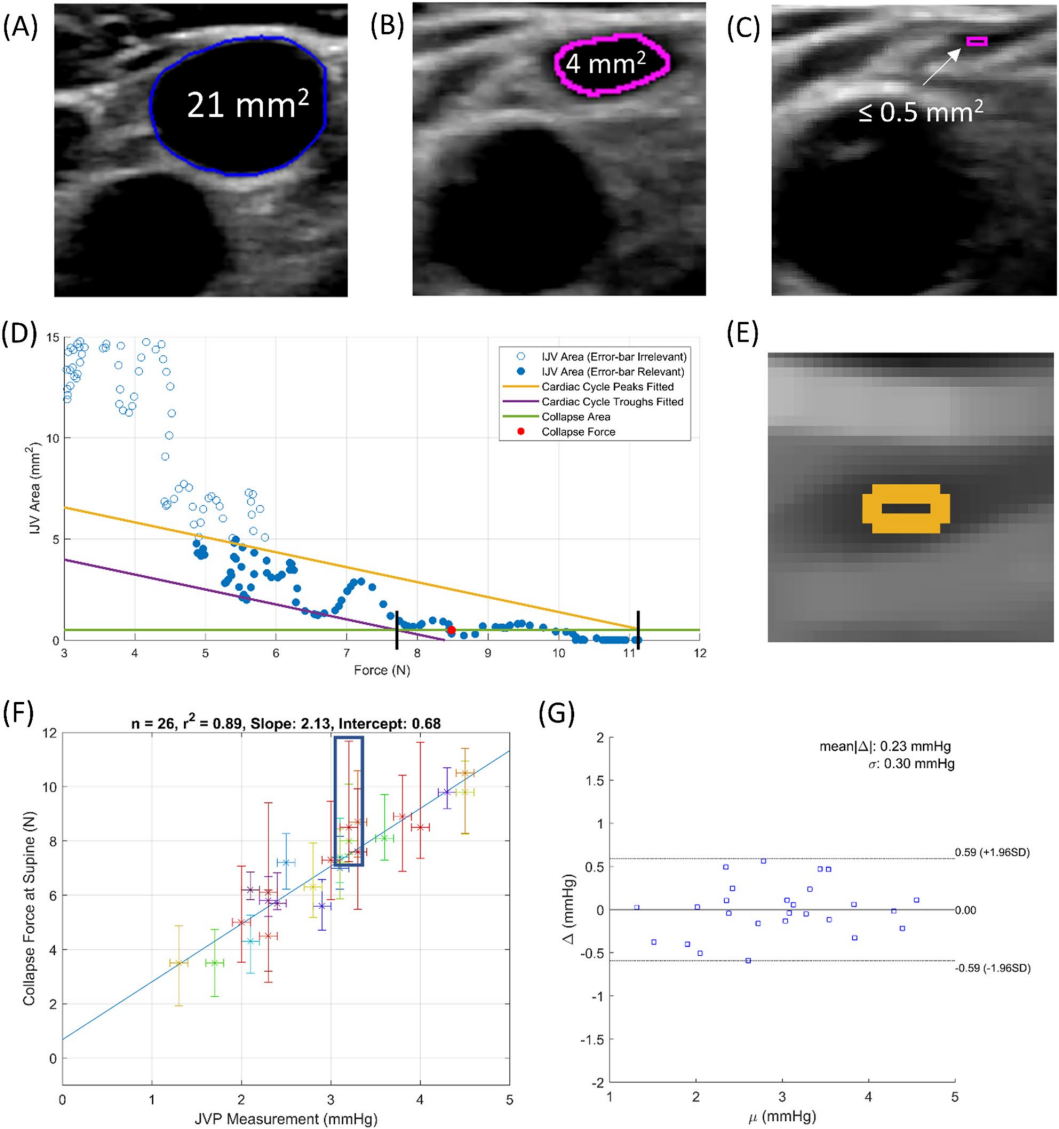
(**A**) Segmentation of an open IJV in subject 21. The full segmentation algorithm is used. (**B**,**C**) Only the first region growing step is used. (**B**) Segmentation of an almost collapsed IJV in subject 21. (**C**) Segmentation of a collapsed IJV in subject 21. (**D**) Collapse force uncertainty contribution due to cardiac cycle in subject 21. Using points with area under 5 mm^2^, lines are drawn to estimate the collapse force at the minimum and maximum points of the cardiac cycle. (**E**) Collapse force uncertainty contribution due to segmentation in subject 21. Uncertainty pixels are shaded in gold. (**F**) Correlation with uncertainty of JVP height and supine collapse force. (**G**) Bland–Altman plot of predicted JVP based on collapse force compared to measured JVP.

Table 1 includes deidentified subject population details. While ensuring that the long axis of the IJV is perpendicular to the compression of the short-axis seen in the ultrasound images, as diagrammed in Supplementary Fig. 2, each IJV is compressed to complete occlusion under various conditions: breathing normally while supine, breathing normally while elevated (negative venous pressure perturbation), and performing the Valsalva maneuver while supine (positive venous pressure perturbation). An example of segmentation during normal breathing supine compression is shown in the force-coupled ultrasound images of Fig. 2A–C. When the IJV is near complete occlusion, the segmentation is simplified to only include the initial region-growing step (Fig. 2B). We define the collapse force to be the force at which the cross-sectional area of the IJV is first below the collapse threshold of 0.5 mm^2^, shown in Fig. 2D with force on the x-axis and Supplementary Fig. 4A with time on the x-axis.

**Table 1:** Subject data population and results. The sixth column is the noninvasive jugular venous pulsation height measurement while the last three columns are the experimental collapse force measurements. Dashes in the last three columns indicate no collapse force is obtained for the given condition and subject.

Subject number	Age (years)	Sex (M/F)	Height (cm)	BMI (kg/m^2^)	JVP (mmHg)	Supine collapse force (N)	16-Deg collapse force (N)	Valsalva collapse force (N)
1	31	M	173	25.1	3.1	7.4	3.6	14.3
2	32	M	168	26.6	2.0	5.0	–	24.7
3	28	M	183	23.7	3.2	8.0	4.1	–
4	30	M	170	21.5	2.1	4.3	3.2	–
5	64	M	180	23.7	4.0	8.5	4.7	17.0
6	26	M	180	20.9	3.6	8.1	5.4	19.4
7	28	M	175	28.1	2.5	7.2	4.2	11.7
8	30	M	175	23.8	1.7	3.5	2.2	12.3
9	72	M	173	25.8	4.5	9.8	–	–
10	26	M	178	23.0	2.3	6.1	4.3	14.6
11	29	M	191	30.0	3.3	7.6	5.1	–
12	30	M	173	25.8	3.1	7.2	4.0	11.6
13	24	M	165	25.8	2.8	6.3	3.7	12.2
14	30	M	173	25.1	4.3	9.8	–	29.9
15	26	F	163	24.0	3.0	7.3	4.1	20.4
16	27	F	163	20.6	3.1	7.0	3.9	10.3
17	25	F	173	17.5	1.3	3.5	2.1	10.6
18	26	M	180	24.4	2.1	6.2	2.4	13.8
19	30	F	165	21.1	3.8	8.9	–	–
20	28	M	170	23.5	4.5	10.5	5.7	19.5
21	28	F	150	21.2	3.2	8.5	2.5	15.8
22	31	F	160	24.3	2.4	5.7	2.6	–
23	30	M	178	22.2	2.3	5.8	–	11.5
24	25	F	157	20.1	3.0	–	3.0	13.9
25	26	M	173	18.2	2.3	4.5	3.8	10.7
26	31	M	175	22.0	2.9	5.6	2.2	21.4
27	29	F	165	21.1	3.3	8.7	4.4	21.8

We quantify the uncertainty in collapse measurement from two sources: (1) variation in IJV area (pressure) based on the right atrium cardiac cycle (Fig. 2D), and (2) segmentation uncertainty (Fig. 2E). On the cardiac cycle uncertainty, when plotting force on the x-axis and IJV area on the y-axis, we do not see a purely monotonic decreasing area curve but rather a monotonic decreasing curve with variation superimposed. This variation stems from the contraction and relaxation of the right atrium during the cardiac cycle causing variation in right atrial pressure and central venous pressure^1^. The collapse force could occur at any point in the cardiac cycle. The segmentation uncertainty is derived from the possible range of pixel coordinates where the true wall could reside based on the region growing pixel intensity threshold and how much force is used to compress those pixels’ area (0.25 mm^2^ at the collapse force threshold). These pixels are highlighted in gold in Fig. 2E. We compare our collapse force measurements in each subject to a quantitative version of the clinical noninvasive standard jugular venous pulsation height (JVP). We assume the distance from the right atrium to the clavicle to be 10 cm and measure the angle of recline when pulsations first start to be visible just above the clavicle. This method is illustrated in Supplementary Fig. 4B. Uncertainty here is quantified via a recent repeatability study at 0.1 mmHg^28^. These are the horizontal error bars in Fig. 2F–G. The vertical error bars represent the sums of the cardiac cycle uncertainties and segmentation uncertainties for each subject.

Figure 2F correlates the measured collapse forces of 26 subjects in the supine position breathing normally to their respective JVP measurements. The linear correlation coefficient is ${r}^{2}=0.89$. We can use the least squares correlation line of best fit to predict JVP from collapse force. In Fig. 2G, we measure the disagreement between the predicted JVP and the measured JVP with an average absolute disagreement of $\mathrm{mean}\left|\Delta \right|=0.23 \mathrm{mmHg}$.

**Figure 3:**
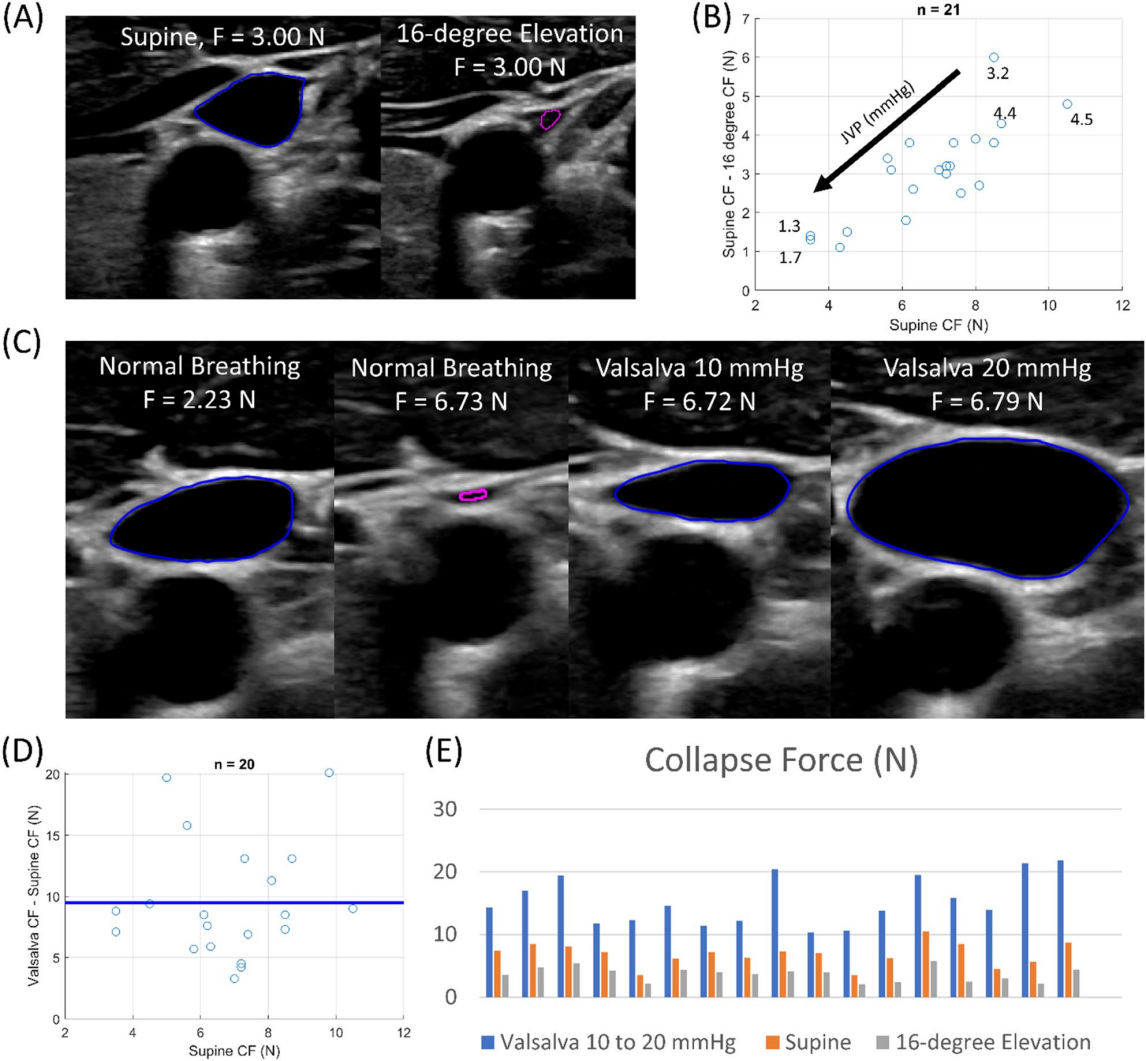
(**A**) Demonstration of hydrostatic effect when elevating a patient from supine to 16° with images at the same force in subject 20. The blue outlines the IJV segmentation while the magenta dot signifies an IJV near collapse. (**B**) Differences between Supine CF and 16° CF in individual subjects. The solid black line signifies the expected effect on collapse force if only hydrostatic effect was present and our linear regression could perfectly predict collapse force from a perfect venous pressure measurement. (**C**) Effects of Valsalva maneuver on increasing IJV area while under force in subject 20. The blue outlines the IJV segmentation while the magenta dot signifies an IJV near collapse. (**D**) Difference between Valsalva collapse force and supine collapse force in the same subjects. The solid blue line represents the mean difference. (**E**) Collapse forces when subjects measure between 10 and 20 mmHg of airway pressure by the Valsalva maneuver compared to collapse forces when subjects are breathing normally while supine and while elevated by 16°.

We next perturb venous pressure in the negative and positive direction. We decrease the pressure in the IJV for each subject by elevating on a tilt table to 16° above supine to decrease hydrostatic pressure. We see in Fig. 3A that the IJV is much more collapsed in the same subject at the same force when the subject is elevated to 16° than when the subject is supine. We note the trend of increasing difference between supine and 16° elevated collapse forces when supine collapse force increases in Fig. 3B. We also note that we cannot measure a collapse force less than 2 N because that is the minimum contact force required for clear images to be obtained.

To perturb venous pressure in the positive direction, simulating decompensated heart failure venous pressures in healthy subjects, we measure airway pressure during the Valsalva maneuver as a proxy for venous pressure^29^. Figure 3C shows the increase in IJV area as a result of increasing airway pressure during the Valsalva maneuver at relatively constant force. This increase in IJV area implies an increase in venous pressure. While measuring airway pressure, we compress the IJV during the Valsalva maneuver at an airway pressure between 10 and 20 mmHg. We consider the difference between Valsalva collapse force and supine collapse force in Fig. 3D. We see that there is virtually no correlation between supine collapse force and this difference, yet there is a wide range of differences. The mean difference signified by the solid blue line is about 9.5 N.

We see in Fig. 3E that for each subject who had data recorded for the supine measurements and each of the two perturbations, the collapse forces during Valsalva while supine are larger than the collapse forces while breathing normally and supine which are larger than the collapse forces while breathing normally and elevated to 16° above supine. We examine the relationships between the perturbed collapse force measurements and JVP in Supplementary Fig. 5.

**Figure 4:**
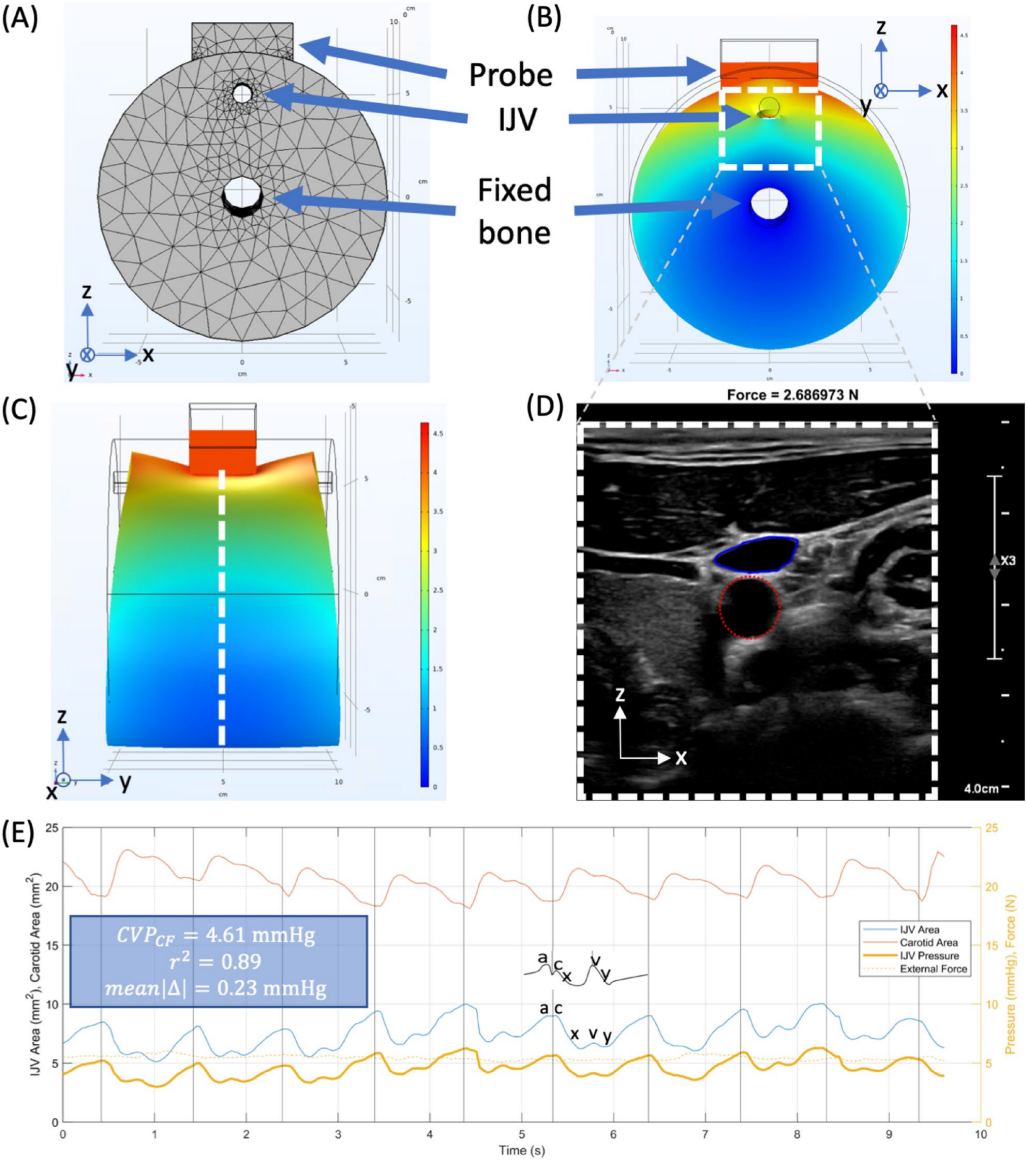
(**A**) 3-D finite element model mesh of the IJV passing through the neck and the force-coupled ultrasound probe contacting the surface of the skin above it. (**B**) 3-D finite element forward model Euclidean displacement (x–z view). The dashed white box is analogous to ultrasound imaging window. (**C**) 3-D finite element forward model Euclidean displacement (y–z view). The dashed white line signifies the ultrasound imaging x–z plane. (**D**) 2-D ultrasound image of segmented IJV (solid blue) and carotid artery (dashed red) short-axis cross-sections for subject 20. (**E**) IJV area, carotid area, IJV pressure estimation, and force plot at an almost constant external force for subject 20. The scalar venous pressure estimate from collapse force is shown with correlation and error data of collapse force and JVP. Black vertical lines symbolize visually-assessed end-diastole from the ultrasound images. A reference central venous pressure waveform from catheterization is overlaid from Tansey et al.^30^. Components of the right atrial pressure waveform reflected in the IJV area waveform are labeled. a: Right atrium (RA) pumps into right ventricle (RV). c: Tricuspid valve closes at systolic onset. x: RA relaxes and tricuspid valve move down. v: RA passively fills; tricuspid opens. y: RA passively empties before pumping.

We utilize frame-by-frame constant force IJV segmentation data, our predicted venous pressure from collapse force, and three-dimensional inverse finite element modeling to produce a venous pressure waveform in the IJV. We first create a triangular prism meshed cylindrical three-dimensional finite element model for the internal jugular vein and surrounding tissue. The vein is modeled as a cylindrical hole in the otherwise uniform Ogden hyperelastic structure. We note a fixed boundary condition at the center of the model to signify the vertebral column, but there is no carotid artery present in the model. A force-coupled ultrasound probe is modeled as a stiff linear elastic material compressing the top of the model over the IJV. In the center of the meshed cylinder is a fixed hole boundary condition to represent the vertebrae. The mesh is depicted in Fig. 4A while the displacement upon compression is shown in Fig. 4B,C. This model is fit to two-dimensional short-axis cross-section force-coupled ultrasound segmented data of the left IJV as it is compressed, as seen in Fig. 4D and demarcated with the white dashed lines in Fig. 4B,C. In our iterative inverse modeling, we set the venous pressure of the average IJV area frame to be the predicted venous pressure from the linear regression of measured collapse force from Fig. 2F,G by tuning forward finite element model parameters. We then arrive at a converged venous pressure for each frame in the sequence via a downhill simplex optimization. For each force-coupled ultrasound frame, venous pressure converges, and we use that pressure as the initial guess for the subsequent frame. We minimize the following cost equation:

1 $$Cost={\Vert {A}_{seg}-{A}_{mod}\Vert }_{2}^{2},$$

where ${A}_{seg}$ is the observed IJV area from segmentation and ${A}_{mod}$ is the IJV area found from running the forward finite element model for a given venous pressure guess. The superscript and subscript 2 indicate we are taking the square of the L2 norm. Figure 4E shows the IJV area waveform, the carotid area waveform, the applied external force, the relevant linear regression results from collapse force measurement to predict CVP, and the venous pressure waveform. We note that the venous pressure waveform has identical morphology to the IJV area waveform. The labeled elements of the waveform (a, c, x, v, y) refer to filling and emptying of the right atrium and opening and closing of the tricuspid valve. Supplementary Fig. 6 details the filtering of the carotid area waveform.

We note that in Fig. 4D, the segmented carotid artery short-axis cross-section lies just below that of the IJV. Furthermore, in Fig. 4E, we note that when the carotid is in systole, the IJV waveform is depressed relative to the CVP waveform reference^30^. When we compare our venous pressure wave estimation for the IJV to a physical model of the transmission of the right atrial pressure waveform to the IJV, we see that all waveform components are present. That said, we note that the physical model has maximum pressure variation in the IJV at the a-wave while our waveform has it at the c-wave. We also note that the v-wave in the physical model’s IJV is not depressed relative to the v-wave in the reference CVP waveform^31,32^.

## Discussion

Our goal in creating a force-coupled ultrasound methodology to estimate lumen pressure in the IJV is to provide a noninvasive, quantitative, and automated approach to venous pressure measurement. Given our high correlation of collapse force with subjects in the supine position breathing normally compared to JVP, we present a method with similar accuracy to JVP in healthy subjects. Like the Korotkoff blood pressure cuff’s ability to measure arterial pressure by measuring the amount of external pressure necessary to collapse a major limb artery, our method yields a collapse force of the IJV proportional to CVP. Given the cuff wraps all the way around the arm, it is able to apply a uniform pressure around the artery of interest. In our case, the force-coupled ultrasound probe provides an external pressure subject to loss such that not all of the external pressure is dedicated towards compressing the IJV. In our narrow, healthy, venous pressure range with the same force-coupled ultrasound, the proportion of loss is predictable. This is likely due to the low pressure of the IJV relative to its anatomical surroundings. Given specifically the supine results, our hypothesis for force-coupled ultrasound collapse force measurement predicting CVP performs well in the relatively narrow range of healthy venous pressures.

Additionally, the collapse force measurement occurs with only two-dimensional information despite using a three-dimensional ultrasound probe and should not require such high pixel resolution to accurately discern collapse force. Hence, to acquire collapse force of the IJV with any vascular commercial ultrasound transducer, a relatively low-cost force-coupling should be constructed for the transducer. However, we note that this collapse force measurement is not a stand-alone measurement yet. Rather, for this study, it is calibrated with JVP measurements. Therefore, the supine collapse force results cannot prove a higher accuracy than JVP in this study. Furthermore, a gold standard comparison to invasive CVP measurement is needed for a full validation of our collapse force methodology and has yet to occur.

When we perturb venous pressure, our experiments yield several noteworthy results. The simplest analysis is that each perturbation of venous pressure produced a concomitant change in collapse force for every subject. Regarding the negative perturbation, we observe a strong trend that as supine collapse force decreases, the difference between supine and 16° collapse force decreases to approach zero. This trend is actually quite intuitive. Collapse forces can only accurately be measured above 2 N while venous pressures around zero can be inferred from hydrostatic offset from supine position and JVP measurement. What’s more, the IJV does not attain pressures below 0 mmHg because it is exposed to atmospheric pressure transmurally, unlike the sagittal sinus which is subdural, and will collapse at 0 mmHg^1^. Thus, as supine venous pressures approach zero, one would expect less of a decrease in venous pressure when raised to 16° above supine.

Regarding the positive perturbation of the Valsalva maneuver, on the one hand, the result gives us confidence that a collapse force will be able to be measured in patients with decompensated heart failure and high venous pressure with our current setup. Hence, none of the venous pressures reached with the Valsalva maneuver were high enough to require a collapse force of more than 30 N. The force-coupling has been shown to translate force entirely and linearly up to at least 45 N while the load cell capacity is 110 N, as addressed in the Supplementary Information. On the other hand, although an increase in collapse force is observed for each subject, the high variability of increase could mean that collapse force is a less reliable predictor of high venous pressures than of venous pressures in a normal healthy range. However, our highly noisy airway pressure measurement makes ascertaining a venous pressure measurement independent from what can be derived with collapse force quite challenging and unreliable. Other sources of variability in our Valsalva collapse force could be different levels of expansion of the IJV downstream toward the superior vena cava yielding variable compliances across the subject population where the IJV is being collapsed and poor Valsalva execution. A more accurate venous pressure measurement at high venous pressures could remove much of the variability observed. Further validation of this method at high venous pressures with a gold standard is of paramount importance to understand its potential impact on heart failure patients.

An assumption for our study is the validity of the JVP measurement which is also reliant on the hydrostatic pressure assumption. When calculating the JVP, one measures the height of the pulsations seen in the IJV in cmH20 and converts to mmHg. This models the IJV as a tube of water and the right atrium as the body of water below it. Our results indicate that the JVP is quite reliable in our healthy subjects given its high correlation to the different collapse force methodology. That said, difficulties in JVP measurement often have to do with the ability to see the IJV pulsations in patient with high BMI or facial hair which includes part of the neck^9,33^. Conversely, collapse force sensing should not be any more difficult in a high BMI patient than in a low BMI patient and facial hair does not obscure ultrasound image clarity given proper acoustic coupling of the probe to the skin. Additionally, JVP methodology has poorer accuracy when estimating high venous pressures such as those observed in decompensated heart failure^9^, which is a major motivation for the development of our collapse force based force-coupled ultrasound method of estimating CVP.

Regarding our three-dimensional inverse finite element optimization to produce a venous pressure wave, we are able to produce a wave which is conceivably accurate, due to the collapse force’s ability to predict JVP (${r}^{2}=0.89, mean\left|\Delta \right|=0.23 \mathrm{mmHg}$), but not directly verifiable. It is highly constrained in that the average venous pressure of the waveform is informed by what is predicted by the collapse force measurement. Additionally, there is a high dependence on the quality of segmentation of the IJV in a B-mode ultrasound image. Errors in segmentation area readily translate to venous pressure waveform errors. Given an accurate segmentation, elements of the right atrial pressure waveform are visible in the venous pressure waveform produced, but they seem to be altered due to the nearby pulsation of the carotid artery, for which our previous study provides a blood pressure estimate^23^. We do not account for the carotid artery in our optimization, which introduces unverifiable error in our IJV pressure waveform, but we can surmise that we are underestimating the v-wave peak in the venous pressure estimate as this occurs during systole. This assumption is corroborated when referring to physical models of the jugular venous pressure wave based on the invasive right atrial pressure wave capture^31,32^. Further investigation with access to central venous pressure or right atrial pressure waveforms from an invasive catheterization is required to examine whether pathological conditions of the right atrium can be accurately inferred from this noninvasively obtained waveform.

In addition to the necessity for further study with comparison against gold standard invasive metrics for venous pressure, there is also reason to make our forward finite element model more accurately reflect observations from the force-coupled ultrasound images other than the IJV. At this point, while producing a realistic but not verifiable amplitude of a jugular venous pressure wave, our three-dimensional forward finite element model with only tracking the IJV is only an overconstrained first step toward producing an absolute venous pressure wave measurement. Utilization of orthogonal plane imaging, as shown in Supplementary Fig. 7, could lead to a more informed estimation of the IJV collapse. What’s more, an increase in complexity of the three-dimensional finite element model to include the carotid artery and a distributed tissue stiffness, informed by observed differential tissue compression in our force-coupled ultrasound images, could allow for our optimization to become significantly less constrained. These improvements could not only provide a more informed optimization for the venous pressure waveform, but also for a carotid artery pressure waveform to be measured simultaneously. Force-coupled ultrasound of the carotid artery has demonstrated potential to provide accurate, rapid, and automated blood pressure measurements^23^. Furthermore, the IJV short-axis cross-section is always visible in the same frame as the carotid short-axis cross-section for all 27 subjects tested. If proven to be accurate and easy to use, force-coupled ultrasound has the potential to provide low cost, noninvasive, and rapid arterial blood pressure measurements while also providing venous pressure measurements.

Here we show that collapse force measurement of the left IJV can estimate venous pressure about as well as JVP in healthy individuals with relatively low venous pressures. We perturb our collapse force measurement to measure lower collapse forces when subjects are elevated to 16°, demonstrating that a hydrostatic pressure decrease is reflected in collapse force. We measure higher collapse forces during the Valsalva maneuver to simulate venous pressures experienced during decompensated heart failure. Finally, we create a venous pressure waveform using force-coupled ultrasound segmentation, collapse force measurement, and three-dimensional finite element inverse optimization. We believe this initial study shows enough promise to merit future clinical studies which include comparison to invasive gold standard direct measurement of CVP via catheterization and further efficacy assessment in heart failure diagnostics and management.

## Methods

### Force coupling construction and signal processing

A force-sensing casing for the Philips XL14-3 xMATRIX ultrasound probe was assembled from 3-D printed from ASA plastic shells (MakerBot Industries, LLC, New York City, New York, United States) and an aluminum frame. Within the casing are an LSB205 S-beam load cell (FUTEK Advanced Sensor Technology, Inc.; Irvine, California, United States) to measure force applied to the ultrasound probe imaging surface and an ADXL335 accelerometer (Analog Devices, Inc., Wilmington, Massachusetts, United States) to provide probe orientation information and account for its effects on force measurement. The load cell signal is amplified by the IAA100 differential amplifier (FUTEK Advanced Sensor Technology, Inc.; Irvine, California, United States), which digitizes, along with the accelerometer signal, into the USB-6001 DAQ (National Instruments; Austin, Texas, United States). More detail on the force coupling is provided in the Supplementary Information.

### Data acquisition

Ultrasound data is observed in real time and recorded on the EPIQ 7C Ultrasound System (Philips; Amsterdam, Netherlands). Force data is observed in real time and recorded with LabVIEW 2016 software (National Instruments; Austin, Texas, United States). Given a new orthostatic position of the subject, the orthogonal incidence angle of the force-coupled ultrasound probe with the long axis of the IJV is noted on the LabVIEW interface in terms of yaw and pitch. This angle of incidence is to be maintained throughout each compression in the given orthostatic position. When recording, three quick compressions are applied to IJV. Then, the user slowly and linearly ramps up force to slightly more than what is necessary for complete occlusion of the IJV. Then, one quick compression is applied. These steps are taken to provide recognizable artifacts in the ultrasound images and the force signal for synchronization later. When the Valsalva maneuver is performed by the subject, the only difference is that the subject does it during only the force sweep using the CR410 digital manometer (EHDIS Car Accessory Co. Ltd.; Guangdong, China) to read the airway pressure applied during the Valsalva.

### Jugular venous pulsation measurement

A subject is slowly tilted downward on a tilt table starting from 60° until the angle where IJV pulsations are visible at the base of the neck. This angle is recorded with an iPhone8 angle sensor app and converted to a hydrostatic pressure by assuming 10 cm between the right atrium and the base of the neck and converting cmH_2_O to mmHg.

### Synchronization of force and ultrasound

Similar to the synchronization of force and ultrasound images for carotid artery force-coupled ultrasound images^23^, we derive an optical flow based position signal from the ultrasound images and use peak detection to find the three quick compressions preceding the force sweep, the force sweep maximum, and the one quick compression succeeding the force sweep. The pairs of first and last peaks are aligned and the three middle peak pairs (two quick compressions and force sweep) are checked to be within the error threshold of an absolute sum of about 0.7 s disagreement. Then a force is assigned to each ultrasound image. All synchronization data processing is done in MATLAB 2021b (The MathWorks, Inc.; Natick, Massachusetts, United States).

### Detection of internal jugular vein

In order to initiate segmentation of the IJV in the force-coupled ultrasound images, the IJV must be detected and a seed point must be provided near the center of the IJV. The IJV is primarily detected via Faster RCNN an object detector neural network, trained on 3000 IJV ultrasound images in a similar manner to the automatic detection of the carotid artery^23^. To avoid the small risk of failure to detect the IJV, the user can click in the IJV when presented with a synchronized force-coupled ultrasound image to initiate segmentation. More detail is provided in the Supplemental Information (Faster RCNN training and results). All IJV detection is done in MATLAB 2021b (The MathWorks, Inc.; Natick, Massachusetts, United States).

### Segmentation of internal jugular vein

When the IJV is not near collapse, region growing from a seed point is followed by a radial line intensity difference maximization, intensity difference and shape optimization, and finally a 2000-point third-order interpolation. The differences from previous work on carotid segmentation are 32 radial lines are drawn after region growing instead of 16 and the 2000-point interpolation replaces the ellipse fit at the end of segmentation^23^. When the IJV is near collapse (area of previous image IJV is less than 5 mm^2^), only region growing is occurs because IJV wall pixels tend to be high-intensity when near collapse such that the region does not grow outside of the walls. Area measurement occurs after the segmentation for a frame is complete by creating a binary image differentiating what is outside the segmentation boundary from what is inside and counting the pixels which are inside.

### Inverse finite element modeling

Creation of the three-dimensional forward finite element model was done using COMSOL Multiphysics software version 5.6 (COMSOL Inc.; Burlington, Massachusetts, United States). Running an inverse optimization involved using COMSOL’s LiveLink functionality with MATLAB (The MathWorks, Inc.; Natick, Massachusetts, United States), allowing MATLAB to run and adjust COMSOL models.

## Supplementary Information

Supplementary Information 1.

Supplementary Information 2.

## Data Availability

In supplementary file cvp_ijv_fcu_rawdata.zip, we provide data from 3 out of our 27 subjects which are already used to provide specific examples in the figures of our manuscript. These subjects are deidentified and numbered 2, 20, and 21. The remainder of the data used during the study to produce our manuscript are available from the corresponding author upon reasonable request.
