# Supplementary material for: Central venous pressure estimation with force-coupled ultrasound of the internal jugular vein
Source: Sci Rep. 2023 Jan 27;13:1500. doi: 10.1038/s41598-022-22867-w (PMC9883282; doi:10.1038/s41598-022-22867-w)
Supplement: Supplementary file 2 — Supplementary Information 2. [file 41598_2022_22867_MOESM2_ESM.pdf]

Central Venous Pressure Estimation with Force-coupled Ultrasound- Supplementary Information

Force-coupling Construction and Force Measurement Error Sources

The force-coupling is custom-made for the Philips XL14-3 xMATRIX ultrasound probe. The mechanical design of the ultrasound probe force measurement adaptor is based on the work of Matthew Gilbertson. Its functional requirements are restated and adapted here as follows:

- Unobtrusive – not impede normal sonographer operation.
- Rapid attachment and removal from ultrasound probe.
- Safe for the sonographer and patient.

As shown in Supplementary Figure 1A, the device is a two-piece, 3D-printed acrylonitrile styrene acrylate (ASA) plastic housing consisting of a base assembly and a magnetically attached cover. The base assembly contains the following components:

- Main frame - provides rigid coupling of the load cell bottom to the plastic base
- Load cell, S-beam type - Futek LSB205 25 lbf (110N) - measures uniaxial force
- Load cell frame - provides rigid coupling of the load cell top to the probe, through the clamping collar
- Clamping collar - a quick-release collar which securely attaches the XL14 probe to the load cell frame
- Accelerometer - Analog Devices ADXL 335, on an Adafruit PCB - for gravity compensation
- Wire cover - to prevent damage to delicate wires during use

The load cell measures uniaxial force along the z-axis as shown in Supplementary Figure 1B. The load cell was selected for its balance of compact size and sufficient overload protection. While the manufacturer specifies a safe axial accidental overload of 1000% of rated output (RO), or 250 lbf (1100N), the probe assembly will be subjected to off-axis forces and moments during use, due to the offset load path and user handling. In this case it is recommended that the maximum combined loading should not routinely exceed the stress value calculated by the manufacturer's equation below.

$$\sigma_{max} \geq 4215 \cdot F_x + 3872 \cdot F_y + 2602 \cdot F_z + 7919 \cdot M_x + 9171 \cdot M_y + 5167 \cdot M_z \quad \text{(S.1)}$$

where forces F and bending moments M are in units of pound force and pound force inch, respectively, and σ_{max} is provided by the manufacturer as:

- 62,000 psi for 10-20 million repeated, reversing loading cycles
- 78,000 psi for 10-20 million repeated, non-reversing loading cycles
- 87,000 psi for static loading

It is reasonable to use the 78,000 psi value as no substantial tensile loading would occur during routine use. Supplementary Table 1 below summarizes anticipated maximum loads during use and their locations. The maximum expected downward force F_z of 30N and small assumed sideways forces F_x and F_y of 1N each give safety factors of 1.1 and 1.0 for static loading and cyclic loading, respectively. Normal operation should fall below this load and cycle range.

Supplementary Table 1: Summary of assumed loads and locations [1].

Variable	Metric Value		Imperial Value		Source
$\boldsymbol{R_x}$	0.022	m	0.87	in	Dimension
$\boldsymbol{R_y}$	0	m	0	in	Dimension
$\boldsymbol{R_z}$	0.041	m	1.62	in	Dimension
$\boldsymbol{F_x}$	1.00	N	0.22	Lbf	Estimate
$\boldsymbol{F_y}$	1.00	N	0.22	Lbf	Estimate
$\boldsymbol{F_z}$	30.0	N	6.74	Lbf	Max. expected
$\boldsymbol{M_x}$	0.041	$N \cdot m$	0.36	$Lbf \cdot in$	$F_y \cdot R_z$
$\boldsymbol{M_y}$	0.706	$N \cdot m$	6.25	$Lbf \cdot in$	$F_z \cdot R_x + F_x \cdot R_z$
$\boldsymbol{M_z}$	0.022	$N \cdot m$	0.20	$Lbf \cdot in$	$F_y \cdot R_x$

The load has an estimated total error ε of 0.15% of its full-scale reading, or 0.17 N (0.038 lbf). This was calculated by the equation below, using manufacturer values for nonlinearity ε_N, hysteresis ε_H, and repeatability ε_R. We do not expect and temperature variation t during use, so zero balance error ε_Z and span error ε_S are neglected.

$$\varepsilon > \sqrt{\varepsilon_N^2 + \varepsilon_H^2 + \varepsilon_R^2 + \left(\frac{\varepsilon_Z \cdot L \cdot N}{W_l} \cdot t\right)^2 + (\varepsilon_S \cdot t)^2} \quad \text{(S.2)}$$

When fully assembled with the ultrasound probe, the force-coupling was calibrated with a combination of calibration weights while fixed in its vertical orientation with a conventional scale with a capacity of 50 lbs and a sensitivity of 0.1 lbs (Accuteck Packaging, Foxboro, Massachusetts, United States). We see in Supplementary Figure 1C, the force transmission to the active site of the load cell is predictable and efficient up to more than the maximum applied force in the study. The scaling factor refers to the average multiple of the force-coupling reading to get the scale reading [1].

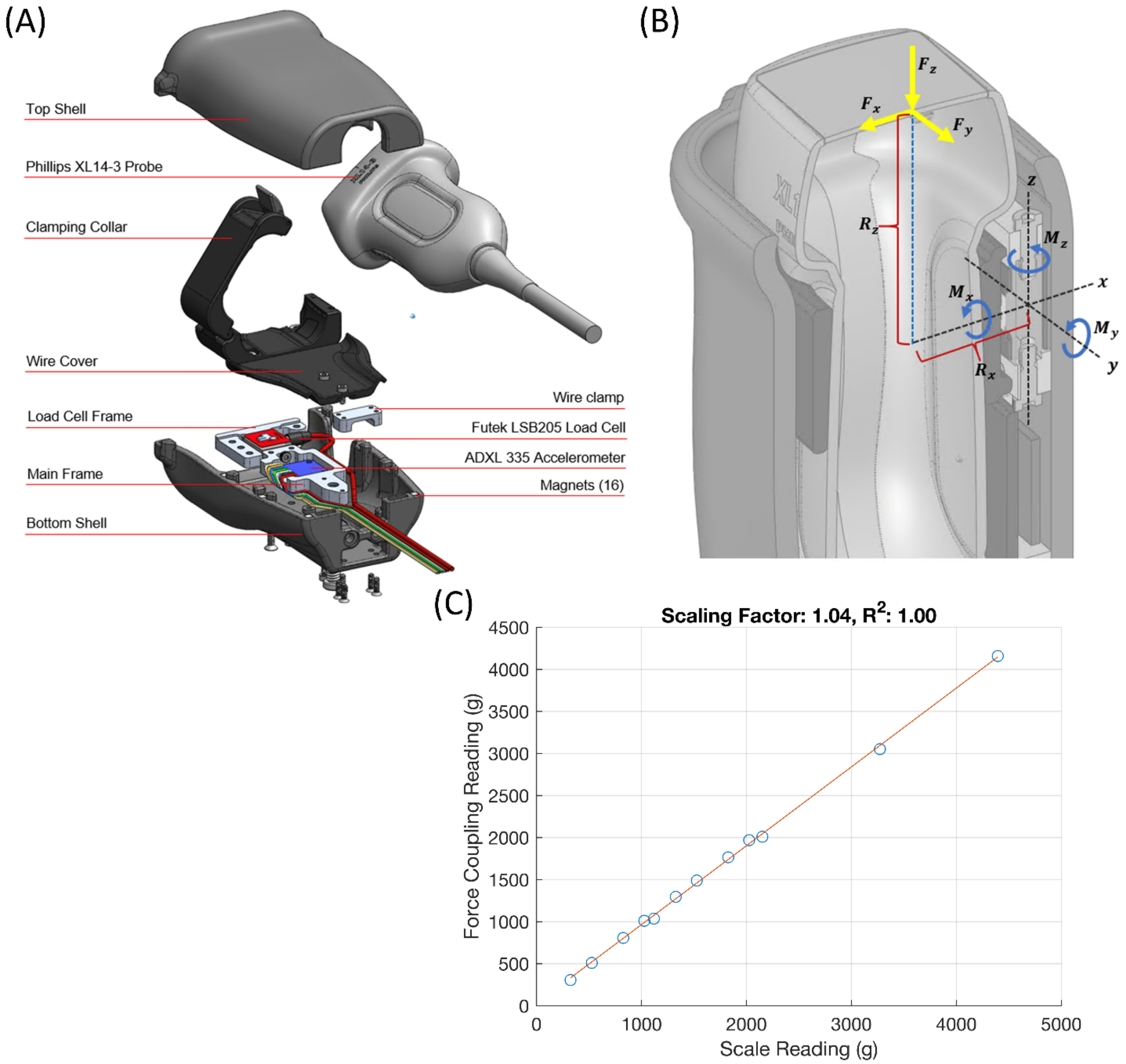

Supplementary Figure 1: (A) CAD diagram of force-coupled ultrasound probe to show individual components. (B) Detailed force translation CAD diagram of force-coupled ultrasound probe. F is force, R is radius, and M is moment. (C) Force-coupling calibration with conventional weight scale.

Orthogonal IJV Compression

When acquiring force-coupled ultrasound images of the short-axis cross-section of the IJV under compression, it is important that the angle of incidence of the probe with the long-axis plane of the IJV is orthogonal. Achieving this will minimize the necessary force to occlude the IJV in comparison to non-orthogonal angles. This angle of incidence can be tracked in the LabVIEW front panel on the system tablet which is shown in Supplementary Figure 2A. When viewing ultrasound images in 3-D mode in the EPIQ 7C system using the XL14-3 probe, one can confirm orthogonality with the long axis plane in two different ways. The first way is by looking at the 3-D rendering which shows white walls in the IJV when walls not at the front of the structure are visible. Seeing these white-rendered walls around the anterior and posterior walls of the IJV means that orthogonality with the long-axis plane is not achieved. The second way is by looking at just the long-axis cross-section. Seeing a completely horizontal long-axis cross-section implies orthogonality with not only the long-axis plane, but also the long-axis itself. Supplementary Figure 2B shows an orthogonal incidence while Supplementary Figure 2C-D are examples of incidences which are not orthogonal.

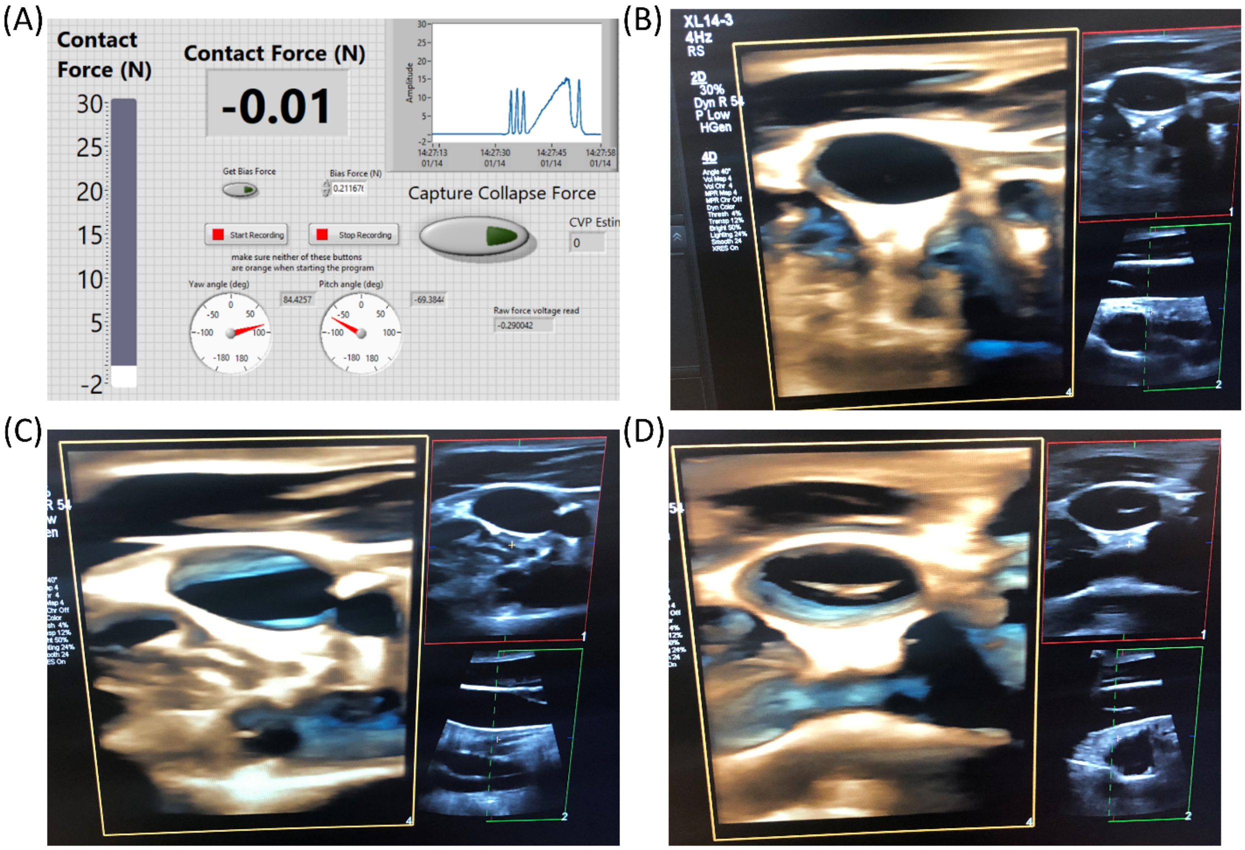

Supplementary Figure 2: (A) LabVIEW front panel showing yaw and pitch angles to be noted when checking orthogonality in 3-D mode and tracked to maintain orthogonality throughout compression. (B) 3-D rendering ultrasound image showing orthogonality of transverse image plane with the long-axis of the internal jugular vein. (C) 3-D rendering ultrasound image showing lack of orthogonality of transverse image plane with the long-axis of the internal jugular vein. Anterior and posterior walls are visible at different points on the long axis in the 3-D rendering. (D) 3-D rendering ultrasound image showing lack of orthogonality of transverse image plane with the long-axis of the internal jugular vein. Long-axis view imaging shows unevenness of long-axis indicating further lack of orthogonality.

Faster R-CNN for Automated IJV Detection

To automate the detection of the IJV in a raw ultrasound image, we train the object detection convolutional neural network Faster R-CNN with segmented ultrasound images of the short-axis cross-section of the IJV. After the detector is trained, it is used to detect the IJV in synchronized force-coupled ultrasound images. A high confidence threshold is used to make incorrect detections exceedingly unlikely. Those which pass the primary search select the correct structure as the IJV in the first frame examined. Those which pass the secondary search select the correct structure in one of the ten frames following the first frame after failing to detect the IJV in the first frame. In the case that neither the primary or secondary search pass, the IJV is detected manually by the user clicking inside the IJV in a displayed ultrasound image to initiate segmentation.

Supplementary Table 2: Summary of automated IJV detection evaluation.

Total	Primary Passed	Primary Pass Percentage (%)	Secondary Passed	Secondary Pass Percentage (%)	Total Failed	Total Pass Percentage (%)	Total Incorrect
128	82	64.1	35	76.1	11	91.4	0

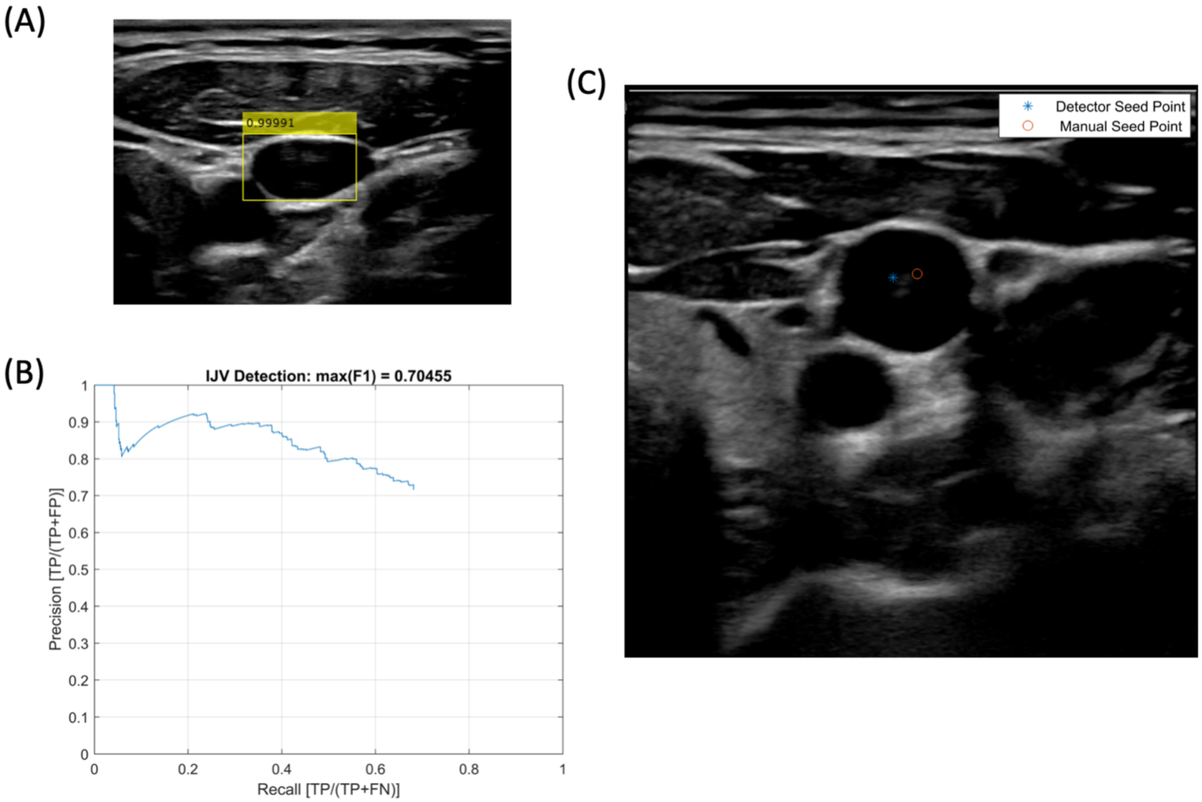

Supplementary Figure 3: (A) Detector bounding box drawn in the vicinity of the IJV with high confidence during detector training. (B) Precision/recall plot for holdout set of carotid detection training data. TP is true positives. FP is false positives. FN is false negatives. (C) Comparing an IJV seed point detected by the carotid detector (blue asterisk) with a centroid derived from the carotid segmentation points (red circle).

Collapse Force Detection and Visual JVP Estimation

As mentioned in the main text, the collapse force is automatically sensed during segmentation as the first frame to reach the collapse force area threshold of 0.5 mm^2. In Supplementary Figure 4A, we track both IJV area and external force as functions of time to add explanation for where the collapse force is sensed before any uncertainty in cardiac cycle or segmentation is quantified. The collapse force in our force-coupled ultrasound method is analogous to quantifying JVP as both seek to estimate venous pressure.

The JVP is most conventionally estimated by measuring the height above the sternal angle. In this study, we aimed to make the method more quantitative by looking for pulsations just above the clavicle while reclining the subject and noting the angle at which pulsations first start to be visible. The precision of this angle is to the single degree, which corresponds to a JVP precision of about 0.1 mmHg after converting from cmH20 to mmHg. For every subject, 10 cm is assumed to be the distance from the center of the right atrium to the pulsation viewing window just above the clavicle. The JVP is calculated from the following equation:

$$JVP = 0.7356 * 10 \sin\theta \tag{S.3}$$

Where the four-digit decimal is the conversion from cmH20 to mmHg, 10 is the assumed distance in cm from the center of the right atrium to the base of the neck, and theta is the largest angle at which IJV pulsations can be seen at the base of the neck.

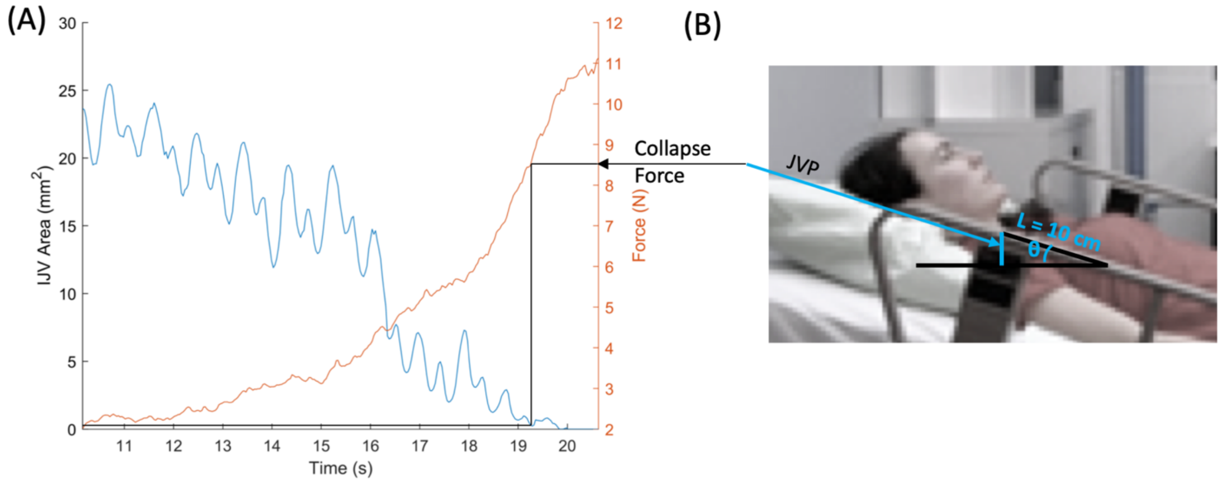

Supplementary Figure 4: (A) Diagram of collapse force derivation. The collapse force threshold is denoted at 0.5 mm^2. A horizontal line is drawn from this area on the left y-axis to where the IJV area curve first intersects. Then a vertical line is drawn from there to the force curve where the collapse force is marked with a horizontal line to the right y-axis. (B) Adapted from https://en.wikipedia.org/wiki/Fowler%27s_position and free to redistribute in any medium or format. Diagram of the jugular venous pulsation (JVP) height derivation. We assume 10 cm between the center of the right atrium and the base of the neck and take the sine of the angle of the visible pulsation in the IJV at the base of the neck to get JVP.

Deming Regressions of Perturbed Collapse Force and JVP

In the main text, a linear least squares line of best fit, which only accounts for uncertainty in the dependent variable, is generated relating supine normal breathing collapse force and JVP measurement because the uncertainty in the collapse force is far greater than the repeatability uncertainty in JVP. However, in the negative and positive perturbations, the uncertainty in the JVP measurement is more equal in magnitude to the uncertainty in collapse force. When elevating the subject to 16 degrees, the JVP measurement is adjusted assuming the distance from the right atrium to the base of the neck is 10 cm to account for the hydrostatic pressure decrease without allowing venous pressures below 0 mmHg. Yet the population of subjects ranges in height, which adds uncertainty to the measurement. Regarding the Valsalva maneuver, a digital manometer measures airway pressure, which is used as a proxy for venous pressure. The uncertainty here stems from the imprecision of the manometer measurement and the indirectness of the airway pressure proxy, yielding an uncertainty of 3 mmHg on each side. Furthermore, the cardiac cycle variation of collapse force decreases because the right atrium is unable to expand and contract during Valsalva.

A Deming regression line accounts for uncertainty in both the dependent and independent variable and is used to produce lines of best fit for the supine normal breathing collapse force and JVP, the 16-degree elevation collapse force and hydrostatic offset adjusted JVP, and the Valsalva collapse force and airway pressure in Supplementary Figure 5A, 5B, and 5C respectively. The r^2 correlation coefficient works to provide a certain level of confidence in the Deming regression lines in that low r^2 should yield low confidence and high r^2 should yield higher confidence [3]. That said, the progressively steeper slopes of the Deming regression lines at higher venous pressures, shown in Supplementary Figure 5D, lends credence to the theory that as venous pressure increases, a smaller percentage of the external force applied is dedicated to collapsing the IJV. Evidence against this theory is also present

given the large gap in assumed venous pressure between the supine and Valsalva Deming regression lines.

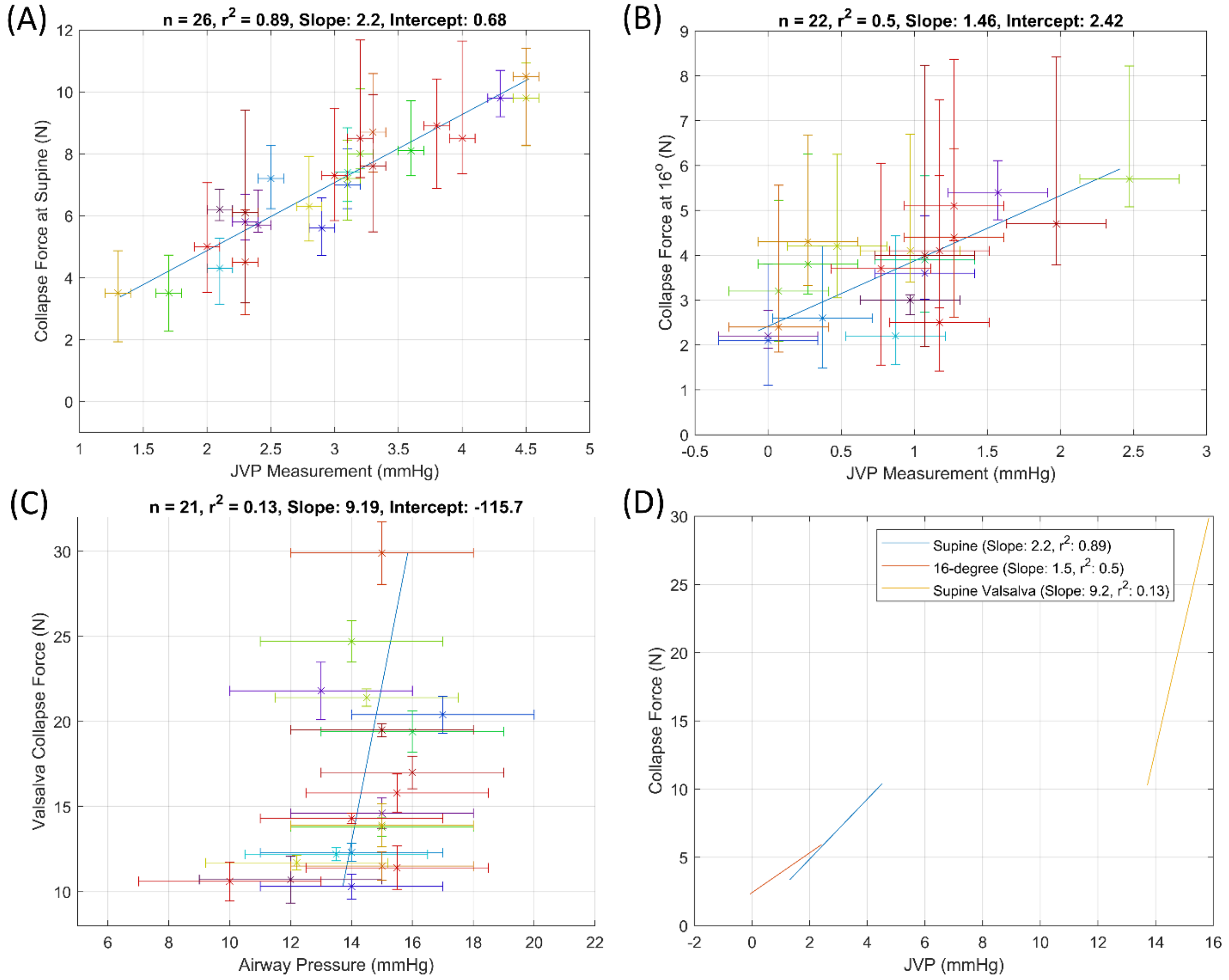

Supplementary Figure 5: (A) Deming regression of supine collapse force with JVP height. (B) Deming regression of 16 degree collapse force with JVP height adjusted for hydrostatic offset. (C) Deming regression of Valsalva collapse force with airway pressure manometer measurements. (D) Combination of Deming regression lines at different collapse forces and inferred JVP measurements.

“Average” Filtering for Noisy Carotid Artery Area Waveform

The carotid artery is fully in the far field of the ultrasound image, which decreases image resolution, yielding a noisy carotid area waveform. An in-band filter is developed in an effort to decrease in-band noise to produce a carotid area waveform of a more typical and consistent morphology in Figure 4E. First, each beat is isolated from the rest of the signal, balanced such that the y-value of the first point in the beat is equal to the y-value in the last point in the beat, interpolated such that the number of samples in the beat is equal to the number of samples in the beat with the most samples, and normalized such that the mean and standard deviation of the beat are zero and one, respectively, to produce an average beat (Supplementary Figure 6A-B). Then, each isolated beat is filtered by comparing each sample of the beat to the analogous sample in the average beat. If the difference is larger than the standard deviation (Supplementary Figure 6C) among the sample at each of the nine beats, the sample in the beat under examination is replaced with the average sample. Supplementary Figure 6D shows an overlay of the raw carotid area waveform and the “average” filtered carotid area waveform. In Figure 4E, the “average” filtered waveform goes through an additional 3-point moving average filter.

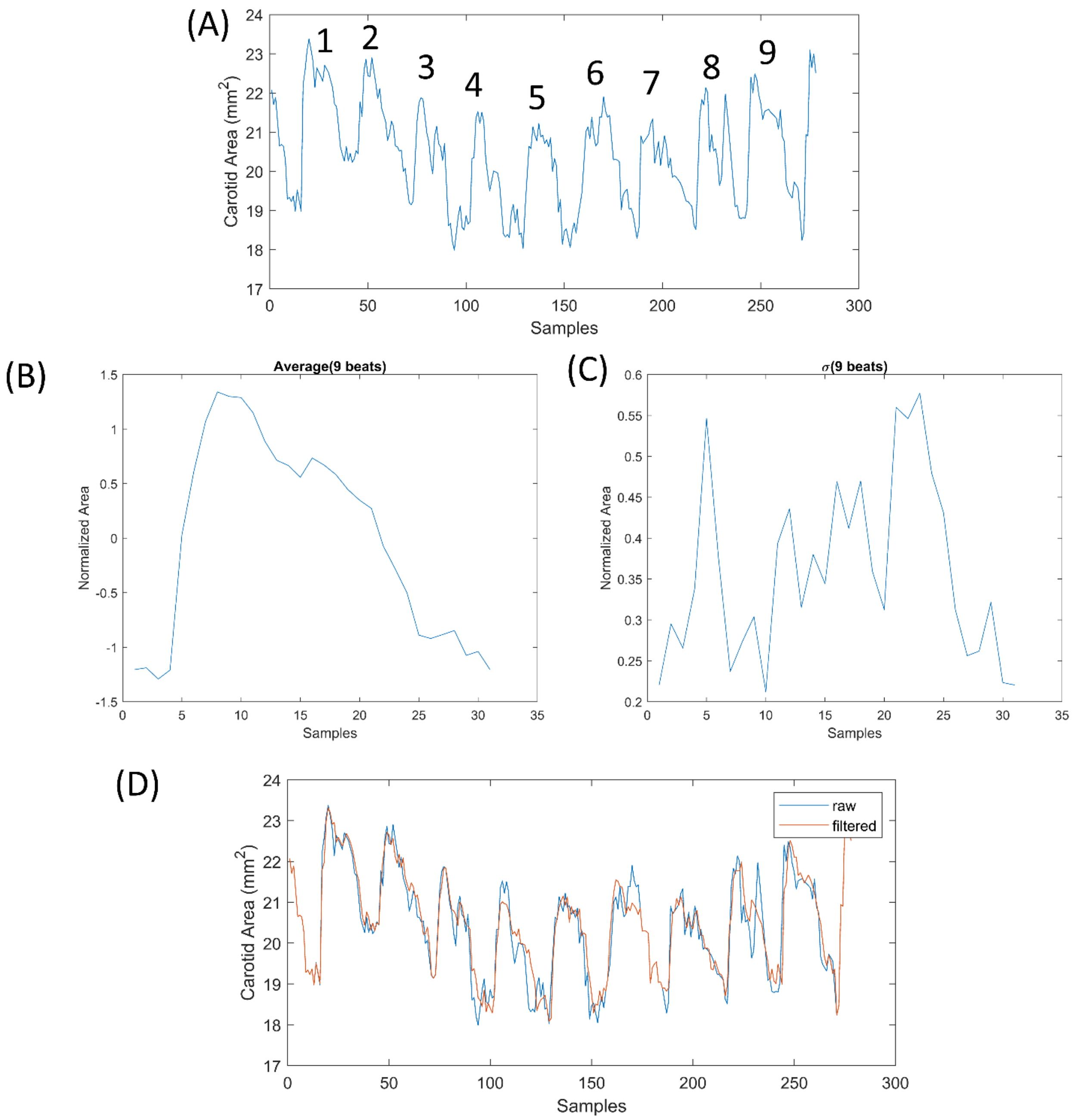

Supplementary Figure 6: Process of "average filter". "Samples" signifies the number of ultrasound frames from which the segmentation was originally derived and is the x-axis for each of the graphs. (A) Raw carotid area waveform with beats numbered. (B) Average beat, normalized in "y" and interpolated in "x". "y" is unitless and normalized. (C) Standard deviation at each normalized and interpolated beat sample. "y" is unitless and normalized. (D) Overlay of raw carotid area and carotid area after going through "average" filter.

Three-dimensional Finite Element Modeling for Orthogonal Plane Imaging

One way of expanding the information input to our three-dimensional forward finite element model when attempting to solve the inverse problem of venous pressure waveform estimation would be utilizing the three-dimensional imaging capabilities. Supplementary Figure 6 illustrates how we would be able to inform pressure with input from two orthogonal planes captured by the XL14-3 xMATRIX probe. This would allow us to compare IJV compression in three dimensions instead of two. In theory, this additional relevant information should lead to a waveform estimate of at least the same accuracy if not higher accuracy than our current two-dimensional observations. The drawbacks are minimal but there are slight losses in frame rate and resolution when viewing ultrasound images in this way. Further exploration of our three-dimensional inverse optimization with the additional long-axis compression observations is an apt next step in improving our venous pressure waveform methodology in addition to the inclusion of the carotid artery pulsation in our forward model.

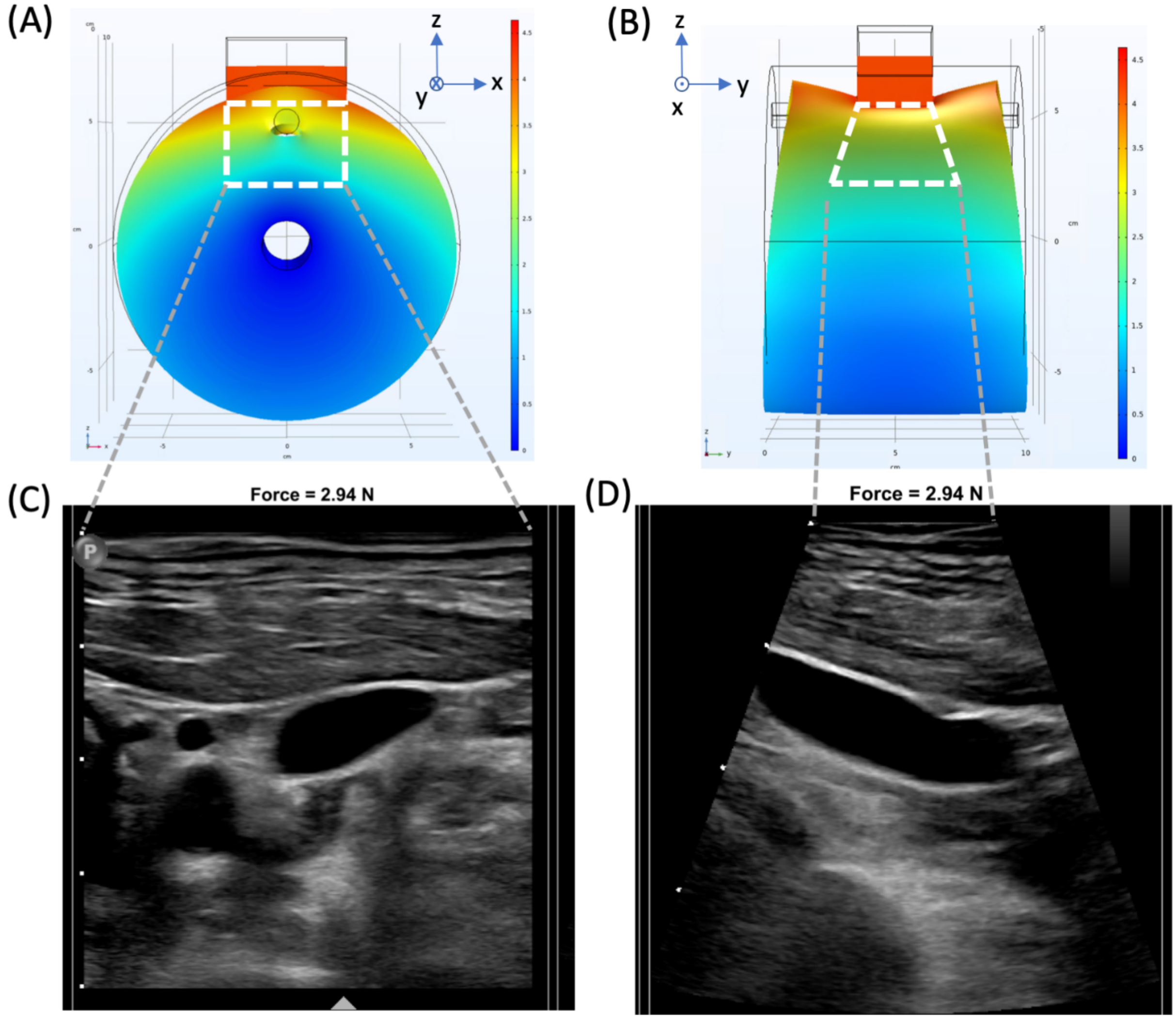

Supplementary Figure 7: (A) Front view of the three-dimensional finite element model displacement result. The dashed white box indicates the image plane viewed in one of the orthogonal ultrasound image planes of the XL14-3 probe. (B) Side view of the three-dimensional finite element model displacement result. The dashed white trapezoid indicates the image plane viewed in the other orthogonal ultrasound images plane of the XL14-3 probe. (C) Force-coupled ultrasound image of the short-axis view of the IJV. Gray dashed lines link this view to the front view of the finite element model. Each dot on the left represents one cm of depth. The gray triangle on the bottom of the image represents the position of the orthogonal plane. (D) Force-coupled ultrasound image of the long-axis view of the IJV. Gray dashed lines link this view to the front view of the finite element model. Each dot on the left represents one cm of depth.

References

[1] "Model #LSB205 Extraneous Load Factors," [Online]. Available: https://media.futek.com/content/futek/files/pdf/extraneous_load_factors/lsb205.pdf.

[2] "Model #LSB205 Specifications," [Online]. Available: https://media.futek.com/content/futek/files/pdf/productdrawings/lsb205.pdf.

[3] R. Martin, "General Deming regression for estimating systematic bias and its confidence interval in method-comparison studies," *Clinical Chemistry,* vol. 46, no. 1, pp. 100-104, 2000.
